# Acclimation of mechanical and hydraulic functions in trees: impact of the thigmomorphogenetic process

**DOI:** 10.3389/fpls.2015.00266

**Published:** 2015-04-22

**Authors:** Eric Badel, Frank W. Ewers, Hervé Cochard, Frank W. Telewski

**Affiliations:** ^1^INRA, UMR 547 PIAF, Clermont-FerrandFrance; ^2^Clermont Université–Université Blaise-Pascal, UMR 547 PIAF, Clermont-FerrandFrance; ^3^Department of Biological Sciences, California State Polytechnic UniversityPomona, CA, USA; ^4^Department of Plant Biology, Michigan State UniversityEast Lansing, MI, USA

**Keywords:** trees, wood, trade-off, thigmomorphogenesis, mechanics, hydraulics, wood anatomy, acclimation to stress

## Abstract

The secondary xylem (wood) of trees mediates several functions including water transport and storage, mechanical support and storage of photosynthates. The optimal structures for each of these functions will most likely differ. The complex structure and function of xylem could lead to trade-offs between conductive efficiency, resistance to embolism, and mechanical strength needed to count for mechanical loading due to gravity and wind. This has been referred to as the trade-off triangle, with the different optimal solutions to the structure/function problems depending on the environmental constraints as well as taxonomic histories. Thus, the optimisation of each function will lead to drastically different anatomical structures. Trees are able to acclimate the internal structure of their trunk and branches according to the stress they experience. These acclimations lead to specific structures that favor the efficiency or the safety of one function but can be antagonistic with other functions. Currently, there are no means to predict the way a tree will acclimate or optimize its internal structure in support of its various functions under differing environmental conditions. In this review, we will focus on the acclimation of xylem anatomy and its resulting mechanical and hydraulic functions to recurrent mechanical strain that usually result from wind-induced thigmomorphogenesis with a special focus on the construction cost and the possible trade-off between wood functions.

## Introduction

Secondary xylem (wood) arises as a result of cell divisions of the vascular cambium and is referred to as a type of secondary growth. After division, these cells go through phases of differentiation, enlargement, and maturation. During this process, the cell wall develops, most significantly, the secondary cell wall composed of organized layers of (crystalline) cellulose microfibrils and lignin. Lignin deposition also occurs in the middle lamella region between cells. At the end of the maturation process, the conducting cells (tracheids in conifers, vessels in angiosperms) die through a process of autolysis, the living components (cytoplasm and organelles) are reabsorbed by the tree, and the water-filled cell lumen becomes physiologically functional providing hydraulic conductivity. The resulting structure is a complex porous network of interconnected cells that fulfills several functions that are required for the continued life of the tree in a variable environment. In both dicotyledonous trees and in conifers the vascular cambium can continue to produce new layers of wood throughout the life of the stem or root and so the response to environmental cues is ongoing.

The xylem of the stem mainly provides three types of functions: (i) the xylem is the hydraulic pathway for the transport of water from the soil to the transpiring leaves, providing hydration to all living cells along the way; (ii) the stem mechanically supports the heavy structure of the tree; (iii) the xylem is a place where many biochemical components that are required for the tree in order to withstand external stresses (like freezing, insect attacks, etc.) are stored as well as the storage of photosynthate (carbohydrates, lipids, and proteins) over the winter for future growth in the subsequent spring (Telewski et al., 1996). Water storage, both on a seasonal and diurnal basis, can also be a crucial function of the xylem (Pratt et al., 2007; Meinzer et al., 2009). The optimal structures for each of these functions will most likely differ. Selection, either natural, via breeding programs or via genetic engineering, to optimize for one function could lead to sub-optimal performance, or even complete failure of another function (Lachenbruch and McCulloh, 2014). For instance, xylem that is highly efficient in water conduction might be so mechanically weak that it could not withstand wind, snow, or ice loading resulting in failure of the stem or branches. In this paper, we will focus on the first two functions of xylem namely hydraulic transport and mechanical support.

As a result of an acclimation process, plants modify their growth when they experience mechanical loading: plants are able to perceive external mechanical stresses that generate the strain of the living tissues (Moulia et al., 2015). These living cells generate signals that engender local or remote molecular responses that modify the wood formation by the way of modifying the cambial activity and the differentiation process (Jaffe et al., 2002; Telewski, 2006; Chehab et al., 2009; Coutand, 2010). The response of plants to mechanically induced flexing, including the brushing or movement of animals against plants, or the flexing of the above ground portions of a plant by wind, ice, or snow loading was defined as thigmomorphogenesis by Jaffe (1973). However, the influence of wind on plants, and specifically trees, was first identified in a study by Knight (1803). Over the course of the ensuing 170 years between Knight’s (1803) and Jaffe’s (1973) publications and subsequently, many studies have been published on the effect of wind on tree growth and morphology (for reviews see Grace, 1977; Jaffe, 1985; Biddington, 1986; Vogel, 1994; Telewski, 1995, 2006, 2012; Mitchell, 1996, 2013; Jaffe et al., 2002; Braam, 2005; Moulia et al., 2006; de Langre, 2008). The most consistent thigmomorphogenetic effects are a reduction in shoot elongation and an increase in radial growth in response to a flexing stimulus resulting in a plant of shorter stature and thicker, stiffer stem. This change in growth results in a change in plant allometry which reduces the effective canopy profile to wind and reduces drag (Telewski and Jaffe, 1986a,b; Rudnicki et al., 2004; Vollsinger et al., 2005; Telewski, 2012).

At the anatomical level, Telewski (1989) described a thigmomorphogenetic acclimation of xylem formation, leading to particular wood structure termed flexure wood. However, the anatomical characterization of flexure wood is still poorly documented. In the same way, very little is known about the direct consequences of the acclimation of the material structure on the mechanical and hydraulic functions of wood formed under mechanical stimuli. Especially, the growth modifications appear to potentially compromise conductive efficiency resulting in a trade-off between the mechanical and hydraulic functions of xylem. Due to the complexity of the interactions between anatomy, hydraulic conductivity, and mechanical strength of wood, there have been few studies addressing all three variables and their corresponding construction cost; especially in the case of acclimation processes like thigmomorphogenesis.

## The Xylem Anatomical Structure

**FIGURE 1:**
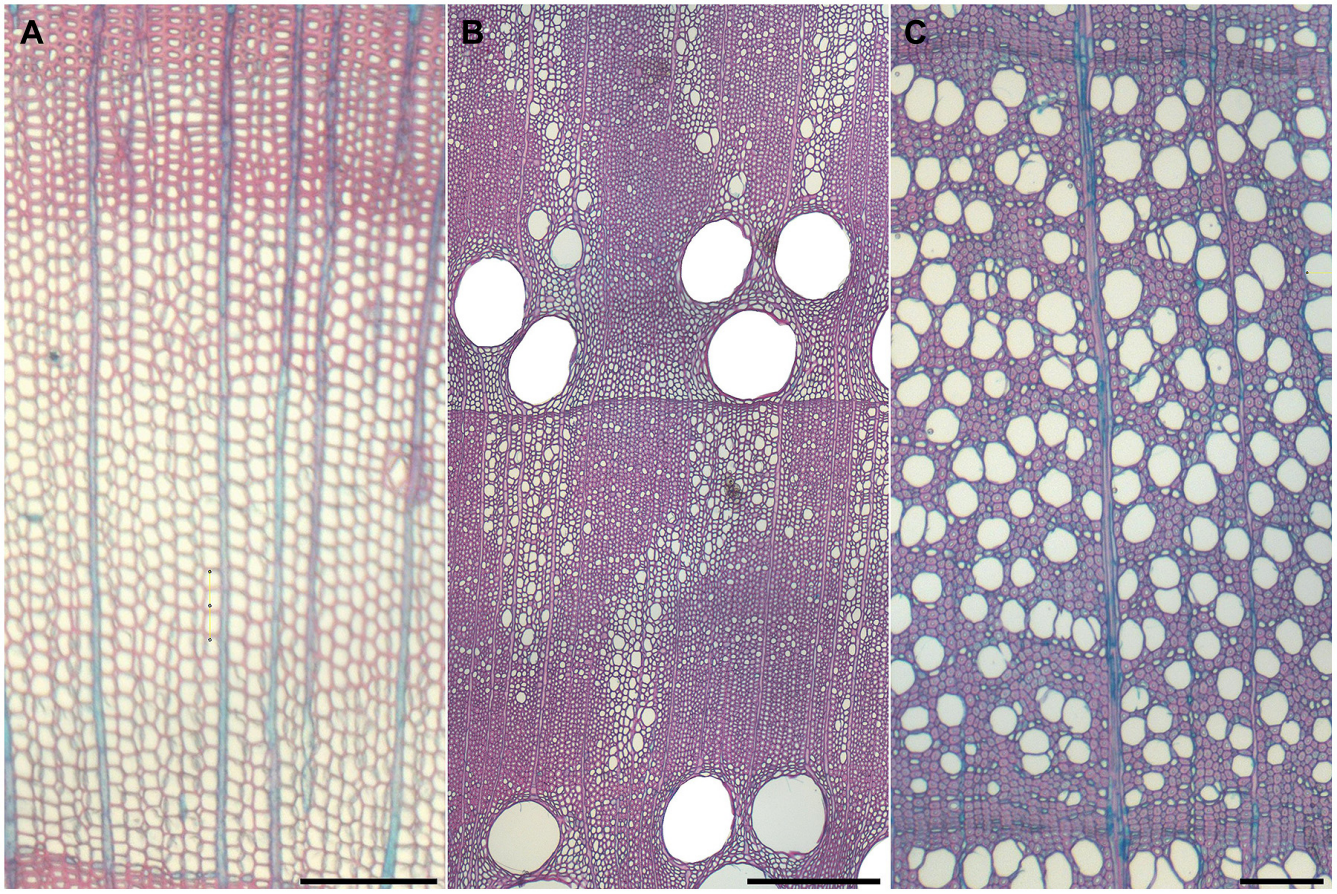
**Typical anatomical cross sections of annual rings in trees.** Gymnosperms show mainly longitudinal tracheids that perform both mechanical and hydraulical functions **(A)**. Angiosperms show heterogeneous **(B)** or homogeneous **(C)** structures that include large vessels that fulfill only the water conduction function, while mechanical support of the tree is provided by fibers. **(A)** *Larix decidua*, **(B)** *Quercus robur*, and **(C)** *Fagus sylvatica*. Scale bar represents 200 μm.

Gymnosperm wood is mainly composed of non-living tracheids with a small portion of living ray parenchyma and when resin ducts are present, living epithelial cells lining the ducts. The earlywood portion of gymnosperms is characterized as large diameter cells with thin cell walls, whereas the latewood is composed of smaller diameter cells with thicker cell walls (**Figure 1A**). The tracheid performs both the conductive and supportive functions within the secondary xylem. The ray parenchyma cells are aligned radially and are usually small compared to angiosperms. On the contrary, the annual ring of angiosperms is composed of more specialized cell types. In addition to ray parenchyma, angiosperms can have axial parenchyma, fibers, fiber tracheids, and vessels depending on the species of tree (**Figures 1B,C**).

Wood density is often used as a key functional trait correlated with other ecophysiological behavior including growth rate, hydraulic conductivity, and mechanical strength (Poorter, 2008; Stegen et al., 2009; Swenson and Weiser, 2010; Swenson et al., 2011; Liu et al., 2012, 2013; Iida et al., 2014). Typically, low density wood results from greater porosities (due to larger diameter, thinner walled cells) that increase the efficiency of water transport, while the material rigidity may be compromised. However, the wood density is an integrative parameter that mainly results from the fiber cell wall thickness and lumen diameter was well as vessel density and size (for angiosperms). It is obvious that for the same mean porosity, different anatomical patterns provide different functional properties.

### Reaction Wood

Trees show a great ability to modify the orientation of their main organs, stem, and branches. They do it in a way that improves their architecture via phototropism or gravitropism, in response to neighbor shading, displacement by the wind, or other mechanical perturbations (avalanches, landslides, slope slippage, ice, and snow, etc.,; for review, see Wilson and Archer, 1977; Timell, 1986a,b,c; Du and Yamamoto, 2007). The dynamic reorientation of these organs involves the formation of a particular type of wood called reaction wood. Usually produced on one side of the organ, the physical and mechanical properties of reaction wood and opposite wood (see for example, Clair et al., 2006) are such as to generate a large difference in growth strains between the sides which can result in a change of curvature of the organ. This wood shows specific anatomical patterns that differ between gymnosperms and angiosperms.

**FIGURE 2:**
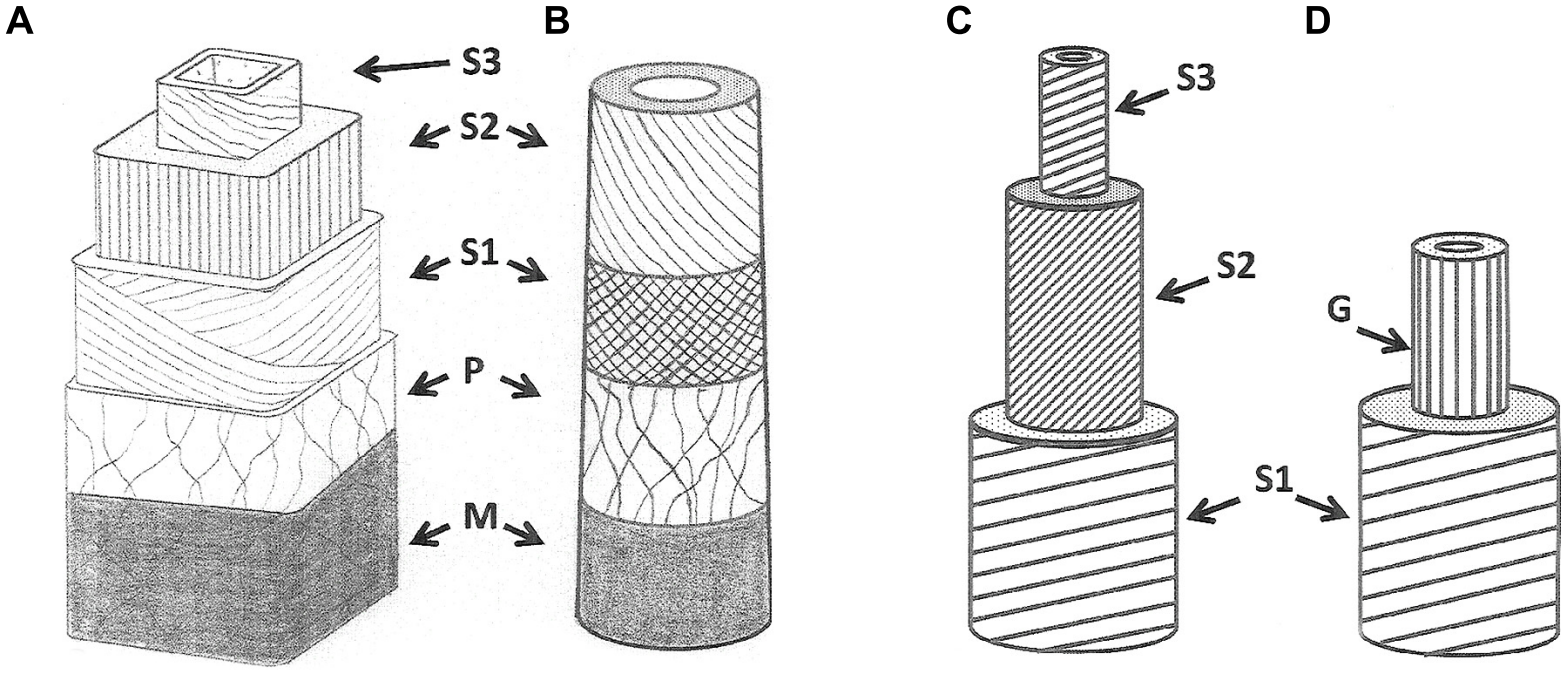
**Normal and reaction wood cell wall structures in gymnosperms **(A,B)** and angiosperms **(C,D)**. (A)** tracheid in normal wood, **(B)** tracheid in compression wood, **(C)** fiber in normal wood, **(D)** fiber in tension wood. G refers to the gelatinous layer.

In gymnosperms, the reaction wood is called compression wood (**Figures 2A,B**), which occurs on the lower side of non-vertical branches. Compression wood tracheids are shorter than normal wood tracheids. In the transversal direction, they are more rounded and show intercellular spaces at would have been cell corners that do not appear in normal wood. At the cell wall level, the compression wood cells usually possess an S3 layer that is not common for normal wood and the thicker S2 layer has a higher microfibril angle (MFA) than normal wood (Evans, 1998).

In porous wood angiosperms, the reaction wood is termed tension wood. The hardwood fibers of tension wood can produce a gelatinous cell wall layer (**Figures 2C,D**), called the G-layer. This G-layer exhibits very particular physical property that generate the differential mechanical states on opposite sides of the organ thus forcing it to modify its curvature via contraction of the tension wood fibers upon maturation. The G-layer is mainly made of cellulose and hemicellulose, has a very small MFA and high degree of crystallinity (Yamamoto et al., 2010).

When trees experience transient wind loadings, the cambium produces a specific wood called “flexure wood” (Telewski, 1989). The anatomy and specific functions of flexure wood are poorly understood. A few observations have been carried out at the tissue level for *Pinus* (Telewski and Jaffe, 1986b) and *Abies* (Telewski, 1989) and by Kern et al. (2005) for poplar. In the conifer *Abies fraseri* the increase in radial growth results from an increase in cell divisions from the vascular cambium, but the tracheid lumens were smaller in size (Telewski, 1989). The angiosperm *Liquidambar styraciflua* exposed to shaking for 30 s once per day exhibited smaller vessel elements in both diameter and length, and fibers were shorter compared to untreated trees (Neel and Harris, 1971). For poplar clones, Kern et al. (2005) reported a significant reduction in vessel lumen area, vessel diameter, and vessel frequency with flexure treatment. Moreover, they observed many similarities between flexure wood and reaction wood. Butterfield and Li (2000) showed that tied trees of *Pinus radiata* produced compression wood and tracheids with lower MFA than non-tied trees. Also at the cell wall level, it has been observed on *P. radiata* that juvenile wood show large MFA in trees growing in open plantations (Cave and Walker, 1994). On the contrary, this MFA is low in established forests, suggesting that wind loadings have a great role in the structure of the S2 layer. In angiosperms, flexing increased the amount of syringyl monolignols over guaiacyl monolignols within the lignin polymer (Koehler and Telewski, 2006). With regard to a functional role for flexure wood, Telewski and Jaffe (1986a) pointed out that flexure wood needs to function in both compression and tension due to alternating sway, and that wood is weaker under compressive loading than tensional loading. Therefore, they suggested that cells with a functional structure more suited to deal with compression would be advantageous to a tree growing in a windy environment. However, the lack of data on flexure wood anatomy, especially at the cell wall level, does not permit a rigorous comparison with reaction wood. More detailed characterizations need to be done before claiming that flexure and reaction woods are structurally the same wood provided by different external mechanical loadings.

### Interconduit Connections

Pits in the double cell wall are the main pathways for water to be transported from one cell to its adjacent cell, connecting two softwood tracheids or hardwood vessels. A hydraulic conduit is connected to multiple other adjacent conduits, providing redundancy in multiple pathways for water movement in case of embolism of one conduit element.

**FIGURE 3:**
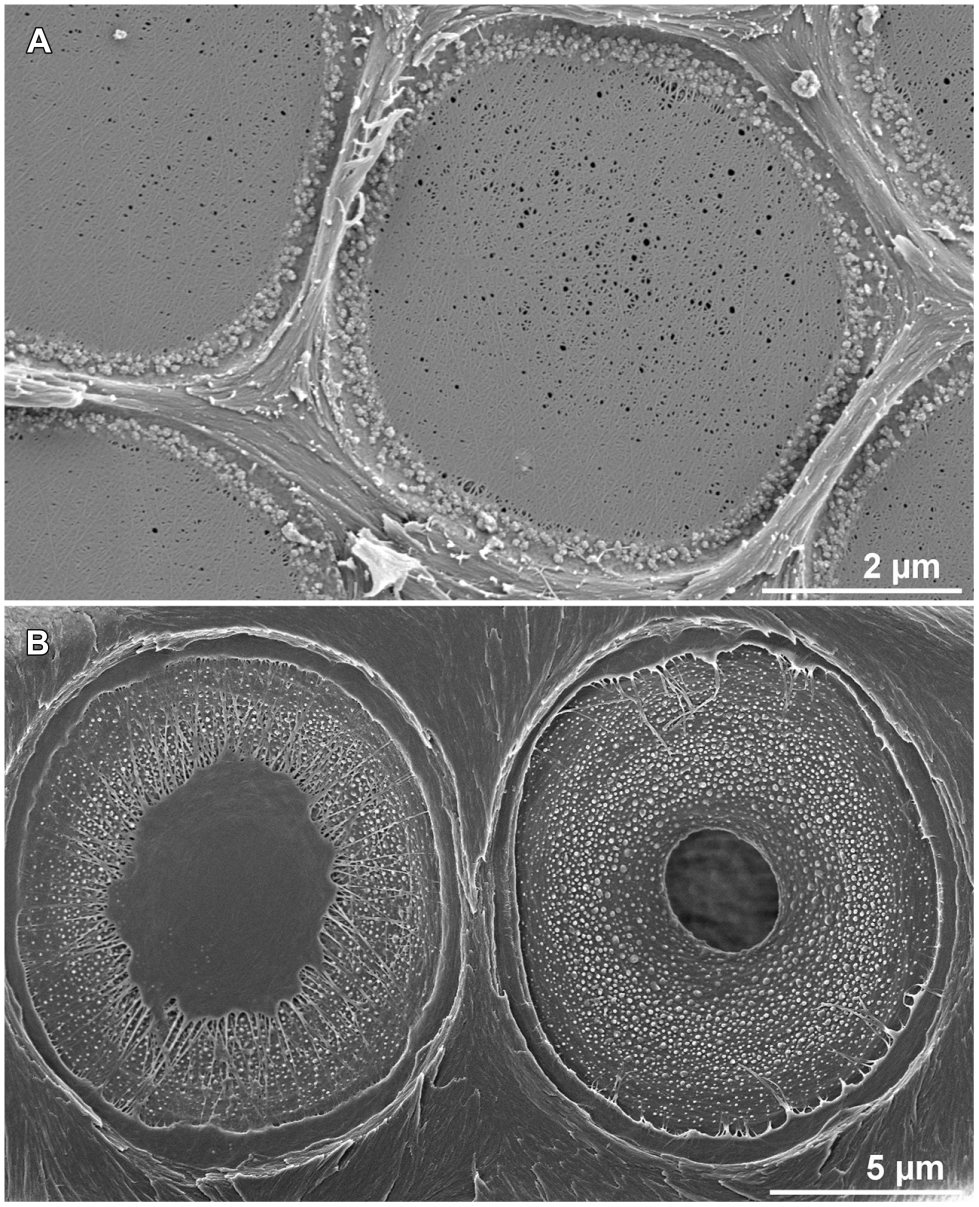
**Structure of pits in angiosperms **(A)** and gymnosperms **(B)**.** The inter-vessel pit in angiosperms is composed of a pit chamber and a thin continuous membrane made of primary wall. Inter-tracheids pits in gymnosperms are made of a pit chamber that includes a thick torus connected to the cell wall by cellulose microfibrils. (photos credit: S. Jansen).

In angiosperms, pits are made of a pit chamber that occurs in the double cell wall and is connected to the cell lumens by the way of holes called apertures (**Figure 3A**). This chamber is separated into two parts by a thin and flexible continuous membrane resulting from the remaining primary wall. In gymnosperms, the membrane is not continuous: the central structure, the torus, is reduced to a central plate; which is physically linked to the cell wall by the margo made of thin threads of cellulose microfibrils (**Figure 3B**).

## Wood Mechanical Behavior

The mechanical functions include: self-support against the pull of gravity in the form of axial compression upon the trunk (McMahon, 1973; Wainwright et al., 1976; Niklas, 1992, 1994), a degree of stiffness or flexibility to bending under windy conditions or under conditions of loading due to ice, snow, or fruit loading (Telewski, 1995, 2006; Valinger et al., 1995; Alméras et al., 2004; Vaast et al., 2005; Mayr et al., 2006) and the ability to generate internal growth strains in developing xylem to produce corrective growth in stems displaced with respect to gravity with the formation of reaction wood. The mechanical parameters that most inform us about significant functional roles in the xylem include the elastic modulus (or Young’s modulus) *E*_*L*_, the modulus of rupture (MOR), and the second moment of cross sectional area *I*, which varies with the fourth power of stem diameter D (Eq. 1). The ecologically important parameter of stem strength is the flexural stiffness *E*_*L*_*I* that drives the ability of an elongated organ to deform when it experiences bending loads.

(1) $$\begin{array}{cc} I=\pi \,\;\frac{D^{4}}{64} & \end{array}$$

The longitudinal Young’s modulus *E*_*L*_ of wood refers to the stiffness of the wood vertically. Since wood is a porous material with longitudinally oriented cells, *E*_*L*_ is mainly linked to porosity (i.e., the cell wall fraction). Thus, in a first approximation, *E*_*L*_ can be estimated as proportional to the wood density (Fournier et al., 2006).

The intrinsic mechanical properties of the cell wall are functions of the cell wall structure (mainly MFA; Cave and Hutt, 1969) and chemical composition (lignin, cellulose, and hemicellulose). For few species (eucalyptus for example) the knowledge of wood density and MFA can explain almost completely the wood Young’s modulus (Evans and Elic, 2001).

Thigmomorphogenesis greatly affects the mechanical behavior of the stem. On the one hand, thigmomorphogenesis modifies the cell differentiation. It results in anatomical changes that decrease *E*_*L*_ of the wood material (Telewski and Jaffe, 1986a,b; Telewski, 1989; Telewski and Pruyn, 1998; Pruyn et al., 2000; Anten et al., 2005; Koehler and Telewski, 2006; Martin et al., 2010). The reduction in *E*_*L*_ is likely a result of increased MFA and not an increase in the syringyl content of the xylem of angiosperms since increasing the syringyl content in transgenic poplar trees increased *E*_*L*_ (Koehler and Telewski, 2006).

The stems of flexed *P. taeda* have a higher MOR compared to non-flexed control trees (Telewski and Jaffe, 1986b), whereas flexing was reported to decrease the MOR of hybrid poplar stems (Kern et al., 2005). This decrease of mechanical behavior cannot be directly explained at the tissue level because the wood density of flexure wood does not decrease (Telewski, 1989). Thus, the physical parameter should be found at the cell wall level and probably involves the MFA in the S2 layer. Telewski (1989) suggested that the reported increase in the MFA of the secondary cell wall of tracheids in *Abies* as part of the thigmomorphogenetic response was responsible for decreasing Young’s modulus.

On the other hand, all the works that dealt with responses to mechanical stimulation of trees reported a large increase in radial growth that increases the second moment of cross sectional area I (Jacobs, 1954; Telewski and Jaffe, 1986a,b; Telewski and Pruyn, 1998; Pruyn et al., 2000; Anten et al., 2005; Martin et al., 2010). This is a consistent response across species and even within half-sib or clonal lines (Telewski and Jaffe, 1986b; Telewski, 1995; Pruyn et al., 2000; Kern et al., 2005). In most cases, the increase in I overrides the decrease in *E*_*L*_ resulting in an increase in stem rigidity or flexural stiffness (*E*_*L*_*I*). The end result is an overall stiffer stem composed of more pliable xylem capable of absorbing more mechanical energy in response to wind loading (Telewski, 1989, 1995, 2012; Pruyn et al., 2000). Moreover, when a circular stem is bent, the maximum longitudinal strain 𝜖_max_ is proportional to the ratio *D*/*E*_*L*_*I* (Eq. 2) and the maximum strain σ_max_ the stem experiences is proportional to the ratio *D*/*I* (Eq. 3).

(2) $$\varepsilon_{\max}=M_{b}\frac{D}{2\,\;\,\;\;E_{L}I}\,$$

(3) $$\sigma_{\max}=M_{b}\frac{D}{2\,\;I}\,$$

Where *M*_b_ is the bending moment applied on the stem (by wind for example). Thus thigmomorphogenesis tends to reduce the strain and stress the stem experiences, making it less likely to fail under mechanical loading.

## Hydraulic Behavior

### Conduction

Trees have developed efficient hydraulic networks to transport water from the roots into the leaves (Sperry, 2003). This process involves small pores (around 20 nm) that generate menisci having a very small radius of curvature, resulting in a surface tension that causes a negative pressure that pulls up the water column from the roots into the leaves, helped by water molecule cohesion.

**FIGURE 4:**
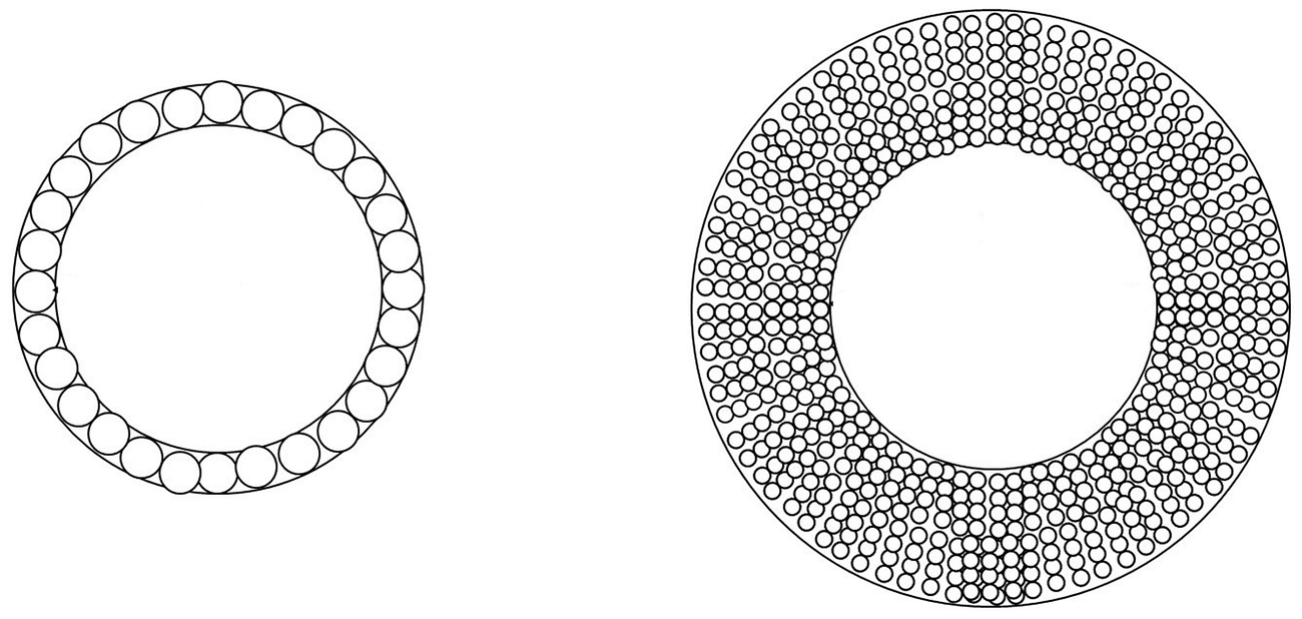
**One ring of large cells are compared with many times smaller cells that provide the same hydraulic conductivity. The mass allocation for the cell wall construction is eight times higher for small cells.** (computed from Tyree and Zimmermann, 2002 and Awad et al., 2012).

Xylem is the main long distance transport pathway for water and soluble mineral nutrients from roots to the leaves. One of its main functions is to provide a low resistance pathway for water transport. Transport is provided by vessel elements (angiosperms) or tracheids (gymnosperms). This water transport does not involve energy consumption by the hydraulic network that act as passive conduits. Conduction efficiency is driven by the Hagen–Poiseuille equation that indicates that hydraulic conductivity *K*s varies with the fourth power (Eq. 4) of the conduit diameter (Zimmermann, 1983). Thus, the optimization of an efficient hydraulic conduction function leads to the construction of a wood structure made of long and very large diameter cells (**Figure 4**).

(4) $$K_{S}\,\;\infty \,\;\underset{xylem}{\Sigma }\,\;d_{h}^{4}\,$$

However, comparing gymnosperm and angiosperm structure, Sperry et al. (2006) clarified the role of the end-wall connections that represented more than 50% of the total resistance of the hydraulic network. They concluded that the conduit length limits the conducting efficiency.

High positive correlations have been observed between water conduction efficiency of the xylem and the growth rate (Tyree et al., 1998; Poorter et al., 2010). This is in accordance with the simple idea that species with fast growth rates usually produce low-density wood that is more porous, with large conduits that are more efficient in transporting water (Eq. 4). Following this correlation, and assuming that thigmomorphogenesis typically generates an increase of the stem diameter growth rate, the flexure wood should show improved hydraulic efficiency. An increase in hydraulic conductivity (reported as sapwood permeability) in free swaying seedlings of *P. elliottii* compared to staked seedlings was reported by Dean (1991). Liu et al. (2002) reported a decrease in specific conductivity despite an increase in radial growth in a thinned stand of *P. contorta* exposed to more wind sway. They concluded the reduction in conductivity was due to sway induced functional damage to sapwood. Kern et al. (2005) reported systematic lower specific conductivity in the stem of different hybrid poplars when they were bent. This local property was mainly due to the formation of smaller vessels as a result of thigmomorphogenesis. However, they reported that the total conductivity of the stem was not affected, suggesting that the increase of wood cross-sectional area due to the thigmomorphogenetic response compensates for the lower local transport efficiency of the xylem.

### Cavitation Resistance

According to the cohesion–tension theory (Dixon, 1914) water ascent in plants takes place in a metastable state under tension. Then, the hydraulic conduction is subjected to transport dysfunctions; drought, and frost stresses can induce very large negative pressure in the water columns that break. This leads to embolism of the conduits that makes it non-operational (Tyree and Zimmermann, 2002). Cavitation is triggered by air entry in hydraulic conduits (Sperry and Tyree, 1988; Cochard et al., 1992). It occurs when the pressure difference between adjacent air- and water-filled xylem conduits becomes large enough to pull the air-water meniscus through inter-conduit pores toward the water filled conduit (Zimmermann, 1983). The required pressure difference Δ*P* is inversely proportional to the diameter of the pores, *d*, according to the Jurin’s law:

(5) $$\Delta P=\frac{4\,\;\tau \,\;\cos\;\theta }{d}\,$$

where τ is the liquid surface tension, 𝜃 is the contact angle of the liquid with the pore walls. Thus, the plant structural parameter that determines the vulnerability to drought-induced xylem embolism is the diameter of the largest inter-vessel pore (Eq. 5).

The main sites of air seeding to the xylem pathway are inter-vessel or inter-tracheids pits. What is the physical role of the pits during the cavitation process? Many anatomical measurements have been performed on gymnosperm and angiosperm pits. There are statistical correlations between anatomical parameters like pit membrane thickness, aperture diameter, torus diameter, etc., with the cavitation sensitivity. The mechanical behavior of the pit membrane was investigated by the way of modeling (Sperry and Hacke, 2004; Tixier et al., 2014). They showed that the pit aperture diameter together with the diameter and the thickness of the pit membrane plays a great role in its mechanical behavior; which was highly correlated with cavitation sensitivity at the inter-specific level. However, no clear mechanism has been described and no anatomical parameter can be clearly said to be the key point. Again, the pit is probably the most relevant level of observation of air-seeding and there is no evidence of relationship with the conduit diameter or length (Dalla-Salda et al., 2014). However, the “rare pit” hypothesis (also named “pit area” hypothesis; Hacke et al., 2001; Wheeler et al., 2005; Pittermann et al., 2006) suggests that the bigger the conduit, the larger its surface and the more pits are located in its wall, thus the greater probability of a defective, wide, or less efficient pit, that could be the air-seeding starting point. Following this hypothesis, cavitation sensitivity may be lower for flexure wood that shows smaller conduit diameters. For gymnosperms, one of the most relevant anatomical parameters that may drive the cavitation resistance is the overlap between the torus and the pit border (torus diameter/aperture diameter). This is consistent with the seal-capillary seeding hypothesis (Bouche et al., 2014). Finally, the xylem anatomy determines how much water can be transported and at the same time, the plant’s vulnerability to transport dysfunctions (the formation and propagation of embolism) associated to water stress.

Despite the cavitation mechanism not being clearly elucidated, several correlations were investigated in order to focus on the relevant anatomical parameters. At the macroscopic scale, the wood density is often investigated and species that show denser wood usually show higher cavitation resistance (Sperry et al., 2006). This is coherent with previous observations that suspected that wider conduits are more vulnerable to cavitation (Carlquist, 1975; Baas, 1986; Cochard and Tyree, 1990; Tyree et al., 1994). However, this interspecific correlation hides a large variability and finally, the relationships between diameter conduits and their vulnerability is probably an indirect and non-causal correlation when it comes to water stress induced embolism. However, freezing induced embolism appears to be fairly well understood and is closely related to the size of the conduit (Sperry et al., 1994; Davis et al., 1999; Pittermann and Sperry, 2003; Mayr et al., 2006). According to these correlations, wood formed under thigmomorphogenetic process should be less prone to freezing induced embolism since the conduits may be reduced in size. However, no work has reported experimental data on the cavitation resistance of flexure wood, suggesting that hydraulic properties need to be investigated in order to confirm these hypotheses.

## Trade-offs

**FIGURE 5:**
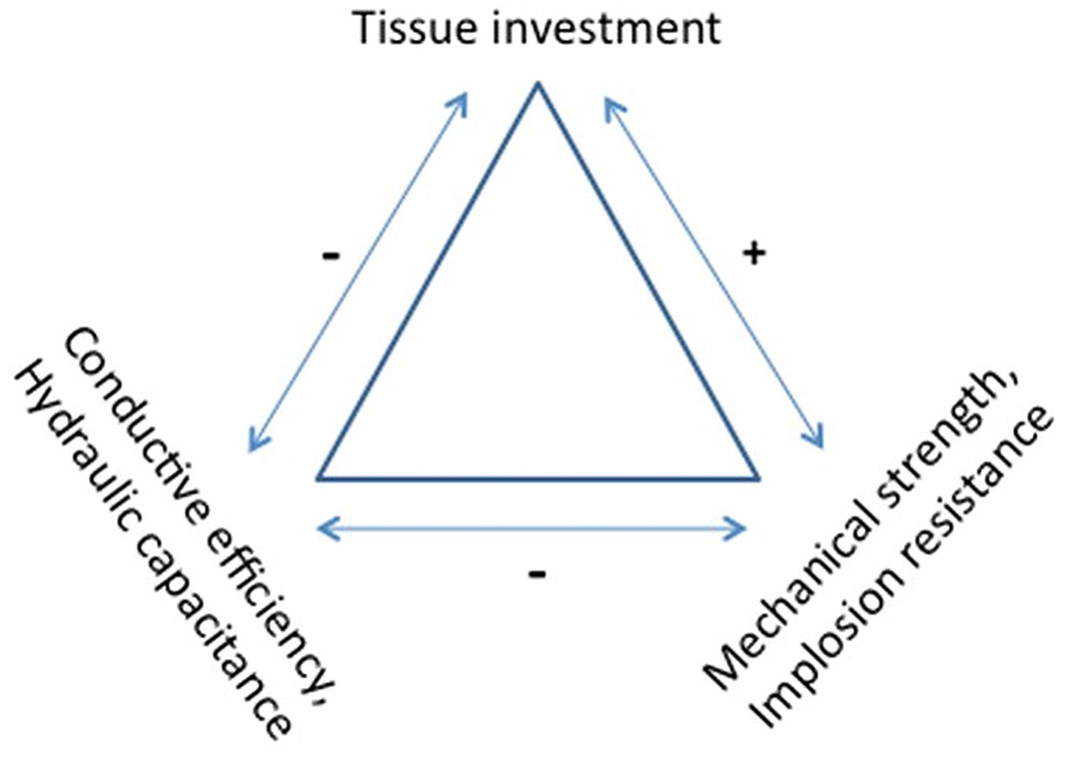
**Functions vs. cost trade-off in wood tissues**.

Considering trees need to continuously manage all their vital hydraulic and vital mechanical functions, it is suspected that trade-offs could exist. Possible trade-offs between mechanical and hydraulic properties are inherently complex since both are subject to fourth power relationships. As noted above, axial stiffness (*E*_L_*I*) is proportional to stem diameter to the fourth power due to the second moment of area calculation, and conductive efficiency is proportional to vessel or tracheid diameter to the fourth power, following the Hagen–Poiseuille law. To increase the hydraulic conductivity the obvious solution is to increase vessel or tracheid diameter, but those solutions could weaken the wood. Is there a necessary trade-off between strength and hydraulic conductivity, and what are the other ramifications of this trade-off? (**Figure 5**). If so, what are the consequences for cavitation and implosion resistance? What is the effect on hydraulic capacitance?

A number of studies have investigated the interrelations between conductive efficiency and mechanical stress acclimation, with inconsistent results. Several studies have found a trade-off between hydraulic conductivity and mechanical strength (Gartner, 1991a,b,c; Wagner et al., 1998; Jagels et al., 2003; Smith and Ennos, 2003; Christensen-Dalsgaard et al., 2007). Others have failed to find any trade-off (Woodrum et al., 2003; Pratt et al., 2007; Rosner et al., 2007, 2008; Utsumi et al., 2010). They suggest that several anatomical variables may confound the influence of the number and diameter of conduits. These variables include pith diameter, ray width, and fiber cell wall thickness, to name a few. Additionally, cell wall structure such as MFA and chemical composition (ratio of lignin to cellulose and lignin monomer composition) are also variables that will influence the mechanical strength of xylem. Inferences can be made based on previous studies. For example, reductions in both tracheid length and vessel element length combined with smaller lumen diameters should increase resistance to conductive flow as well as reduce the volume of water conducted on a per conductive element basis. This may be compensated for by an increase in the number of conductive elements, as is the case in conifers, but may be less likely in angiosperms as the total number of vessels also appears to be reduced in thigmomorphogenetic response to wind sway or flexing. Woodrum et al. (2003) investigated the interspecific relationship between anatomy, mechanical properties and water transport of *Acer* but they observed no trade-off between *K*_s_ max and MOE or MOR across the genus. Although compression wood has narrower tracheids and lower conductivity (Spicer and Gartner, 1998) and lower cavitation resistance (Mayr and Cochard, 2003) than opposite wood, in angiosperms tension wood may be similar in conductivity and cavitation resistance as opposite wood (Gartner et al., 2003). Unfortunately there is no published data for cavitation resistance in flexure wood except Kern et al. (2005) who reported that mechanical flexure increased stem rigidity, reduced the number and diameter of vessels, and significantly reduced *K*_S_. However, the treatment did not significantly impact whole-stem K_h_ or the percent loss of conductivity due to embolism, suggesting the lack of hydraulic-mechanical trade-off during mechanical acclimation.

Although the existence of a trade-off between conductive efficiency and resistance to cavitation or implosion (efficiency versus safety) has not been consistently reported in the literature (Cochard, 1992; Sperry et al., 1994), available evidence suggests at least a weak negative correlation (Tyree et al., 1994). The hydraulic efficiency versus safety trade-off was reported by many authors (Martinez-Vilalta et al., 2002; Choat et al., 2005). Martinez-Vilalta et al. (2002) reported for nine co-occuring species that the relationship between specific hydraulic conductivity (*K*_s_) and resistance to cavitation followed a power function with exponent ≈-2, consistent with the existence of a trade-off between conductivity and security in the xylem. However, they suggested that this relationship was consistent with a linear relationship between vessel diameter and the size of inter-vessel pores, which has never been demonstrated. For instance, Choat et al. (2003, 2005) reported trade-offs between conductive efficiency and resistance to cavitation in seasonally dry rainforest trees, but they found no evidence of differences in pit membrane porosities in those species (Choat et al., 2003). Therefore the inverse relationship between water-stress embolism and conductive efficiency is probably indirect (see pit area hypothesis above) whereas the inverse relationship with freezing-induced embolism appears to be direct, as the size of the conduit is directly related to freeze-thaw embolism.

Xylem safety (resistance to cavitation or conduit implosion) and mechanical strength have been found to be positively correlated (Jacobsen et al., 2005; Pratt et al., 2007; Rosner et al., 2008; Utsumi et al., 2010; Bouche et al., 2014). Hacke et al. (2001) defined a mechanical safety factor that evaluates the resistance of a theoretical 2D regular cellular structure to bulk in the transverse direction when submitted to negative pressure. It involves the lumen diameter (*b*) and the cell wall thickness (*t*). The higher the thickness to span ratio (*t*/*b*)^2^, the greater the resistant to implosion. This safety factor was then used for evaluating the implosion resistance of vessels. All these works suggest that wood density, which is highly correlated with conduit size and cell wall thickness, may impact the both functions in the same way, with thicker cell walls that make the tissues stiffer and more mechanically resistant (Hacke et al., 2001). However, smaller lumens impair the hydraulic conduction. According to Pratt et al. (2007), the stem mechanical strength appears to be important in maintaining xylem transport under negative pressure and this could be a strategy both to prevent vessel collapse and to withstand mechanical stresses caused by gravity or wind. However, Bouche et al. (2014) recently argued for angiosperms that the implosion process is unrealistic since most of the species do not experience the pressure level that should be involved. Note that for angiosperms, the implosion resistance is usually computed using the vessel wall thickness and the vessel diameter, since implosion resistance of an isolated vessel probably depends essentially on the transversal rigidity of the surrounding tissues.

Jacobsen et al. (2005) suggested the hypothesis that fibers may be essential for enabling the vessels to achieve great sizes: larger cell wall thickness of surrounding fiber tissues probably provides better implosion resistance with a cost of carbon allocation that is observed in the wood density data (Sperry et al., 2006). Following this consideration, the angiosperm species are probably able to adjust the fiber formation around the vessel in order to avoid vessel implosion (Jacobsen et al., 2005). But perhaps that would be more expensive than putting more cellulose and lignin into the vessel cell wall. Turner and Somerville (1997) showed vessel implosion in an *Arabidopsis* mutant deficient in the deposition of secondary cell wall cellulose. Jones et al. (2001) reported that a 50% reduction in lignin content also resulted in collapsing vessels in *Arabidopsis*. Genetically modified hybrid poplar trees (*Populus alba* × *Populus grandidentata*) with reduced cell wall lignin have also shown collapsed xylem (Coleman et al., 2008). In a global study of 3005 angiosperm species, vessel diameter was strongly linked to conductive efficiency, but not linked to overall xylem density. The fiber tissues are extremely variable and may compensate for weaknesses that large vessel lumen areas can present (Zanne et al., 2010).

A significant but less studied relationship is the positive correlation between conductive efficiency and hydraulic capacitance of the sapwood. This relationship has been found in a wide range of conifers and angiosperms (Choat et al., 2005; Domec et al., 2006; Pratt et al., 2007; Meinzer et al., 2009). At least in the Rhamnaceae, hydraulic capacitance was not related to the sapwood parenchyma area (Pratt et al., 2007) Species with high conductive efficiency apparently have greater stores of apoplastic water in fibers as well as in tracheids and vessel lumens. In one model it is shown that embolism of vessels could contribute significantly to hydraulic capacitance on a daily or seasonal basis (Hölttä et al., 2009). Here again, because of a dramatic lack of experimental data, the impact of thigmomorphogenetic response on the balance between hydraulic and mechanical functions is a virgin field of research that needs to be investigated in order to evaluate the possible impact of deformation due to windy conditions on the hydraulic efficiency and safety.

## Function vs. Tissue Investment

Where are the costs needed in order to improve the functional properties? As seen previously, there are two ways to increase the flexural rigidity *E*_L_*I* of the stem: increasing the elastic modulus of wood or increasing the stem diameter. At the cell level, increasing the module of elasticity usually involves a carbon allocation cost in order to increase the cell wall thickness and to reduce the material porosity (Larjavaara, 2010). Another mechanism to increase the Young’s modulus is to decrease the MFA in the S2 layer of the cell way. Modifying the MFA probably does not involve an energetic cost, but it could impact the dimension of the strengthening. While a low MFA increases Young’s modulus, it might also decrease hoop strength and cause a conduit to become more prone to implosion. The formation of cellulosic G-layer in angiosperm, provides an higher stiffness to the cell but again involves a large amount of cellulose and definitely an additional construction cost in term of carbon allocation.

**FIGURE 6:**
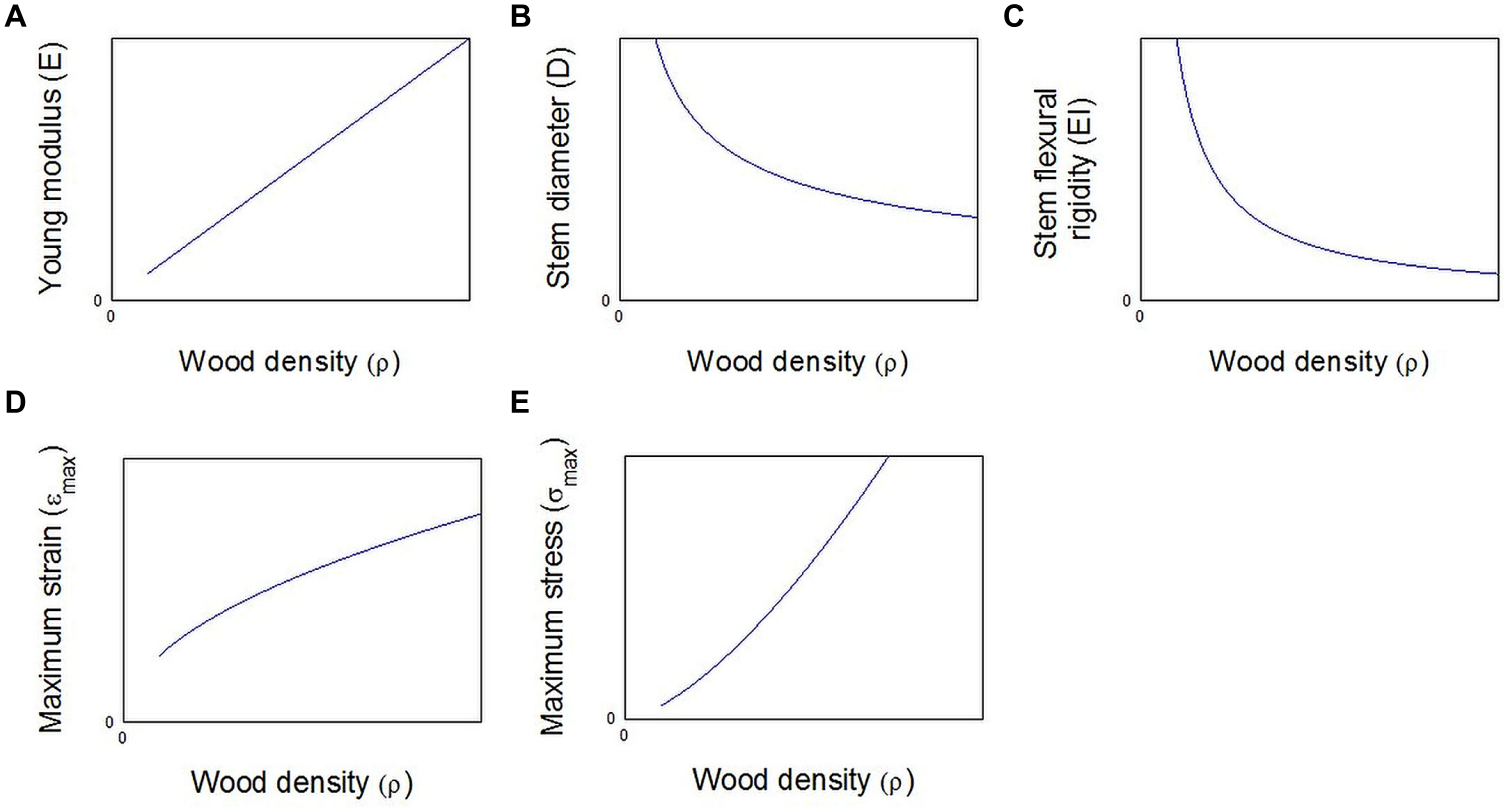
**Mechanical parameters as functions of wood density for a constant biomass *m* in the stem cross section. (A)** The Young modulus is computed according to the Eq. 6. **(B)** The stem diameter is computed according to Eq. 8 and **(C)** the stem flexural rigidity is computed according to Eqs 1, 6, and 8. **(D)** Maximum strain and **(E)** maximum stress are computed for the same fixed external load, respectively, according to Eqs 9 and 10.

At the trunk level, increasing the organ size is an efficient option too. Awad et al. (2012) stated that for the same amount of cell wall, it is more efficient in terms of stem flexural rigidity *E*_L_*I* to add large cells with thin cell walls than adding small cells with thick cell walls. The lower elastic modulus of the wood material is clearly counterbalanced by the large increase in the second moment of cross sectional area I of the trunk (**Figure 6C**). This can be easily demonstrated assuming that the Young’s modulus is proportional to the wood density ρ (Fournier et al., 2006; **Figure 6A**)

(6) $$E_{L}=\alpha \,\;\rho \,$$

When a beam experiences a bending moment Mb, the Eq. 2 tell us that the maximum strain varies as the inverse of *D*^3^.

(7) $$\varepsilon_{\max}=M_{b}\frac{D}{{2\,\;E}_{L}I}=M_{b}\frac{32}{\pi \,\;\;E_{L}\,\;D^{3}\;\;}\,$$

Assuming the allocated biomass m can be estimated as (**Figure 6B**)

(8) $$m=\rho \,\;D^{2}\,$$

Merging Eqs 6, 7, and 8, we can write that the strain varies as the square root of the density and the maximum strain varies according to ρ^3/2^ (**Figures 6D,E**):

(9) $$\varepsilon_{\max}=M_{b}\frac{32\sqrt{\rho }}{\alpha \,\;\pi \,\;\,m^{3/2}}\,$$

(10) $$\sigma_{\max}=M_{b}\frac{32\,\;\rho^{3/2}}{\pi \,\;m^{3/2}}\,$$

These Eqs 9 and 10 demonstrate that, for the same biomass allocation m, the mechanical strain and stress increase with the wood density and put the trunk at risk. This conclusion is still available even if we consider that the critical stress increases in a proportional way to the wood density (Chave et al., 2009). Finally, from a pure mechanical point of view, the formation of annual rings made of large cells with thin cell walls is probably a better strategy than the building of small annual rings made of small cells showing thick cell wall, even if locally the mechanical property *E*_*L*_ is higher in this last case (**Figure 5**).

As seen previously, the optimization of conduction properties and biomass allocation leads to large cells with thin cell walls. Thus, finally, low density wood that includes large lumens would be the best way to manage both mechanical and conduction properties. But what is the cost for building cavitation resistant xylem tissue? The pit is probably the very relevant level of observation. This suggests that building resistant pits has a cost and should take account in maintaining the conduction efficiency. And what is the relative importance of resistant pit members to prevent air seeding, versus mechanical support by walls of tracheids or fibers, to prevent implosion? If both parameters are relevant, and have co-evolved, this could explain some contradictions in the literature. The existence or otherwise of functional trade-offs in wood structure is still unclear and has been debated for the past several years. The anatomical parameters that drive the mechanical properties are now well identified and most of the drivers of the conduction efficiency are identified too. But the way the trees build wood that is resistant to cavitation is still a key question that needs to be elucidated in the next years.

## Conclusion

Trees have many ways, at the cell wall level or at the organ level, to acclimate their xylem structure to recurrent mechanical stimuli (**Table 1**). The acclimation process differs between angiosperms and gymnosperms and the consequences on the hydraulic and mechanical properties of wood are highly variable. Because of the lack of experimental data, there is a crucial need for new investigations in order to characterize the mechanical and hydraulic properties of flexure wood. Moreover, in many windy regions, wind is often directional. Hence, bending occurs in a non-symmetric way, with the leeward part of the stem experiencing more compression stress and the windward portion experience more tension stress. Thus, what is really flexure wood? We suggest to the need to investigate separately the thigmomorphogenetic response of wood formation of both elementary stress (compression or tension) to better understand the acclimation process. On the one hand, there is a real need to investigate the relationships between the mechanical stimuli and the modifications of the anatomical structure and its associated construction cost. For this task, there is a real need to investigate the wood formation process, including at the level of cell division and the cell differentiation. This also requires new studies regarding the molecular mechanisms that link mechanical perception to the mechanical stress induced wood formation.

**Table 1:** Comparison of flexure wood features with normal wood in conifers and angiosperms.

Flexure wood anatomy and functions	Angiosperms	Gymnosperms
Radial increment	+	+
Wood density	0	+
Vessels density (nb vessel/mm^2^)	-	NA
Vessel diameter	-	NA
Fiber length	-	NA
Tracheid diameter	NA	-
Tracheid length	NA	-
Microfibril angle (MFA)	+	+
Module of elasticity (*E_L_*)	-	-
Modulus of rupture (MOR)	-	+
Second moment of cross sectional area (*I*)	+	+
Flexural rigidity (*E*_*L*_*I*)	+	+
Specific conductivity (*Ks*)	+	-
Total conductivity (*K*)	0	0
Cavitation resistance	NA	NA

“+” indicates the increase, “-” indicates the decrease, and “0” indicates no change. “NA” refers to non-available data.

On the other hand, we need to characterize the consequences of the thigmomorphogenetic process on the mechanical properties that help the trees to be better acclimated to further mechanical stimuli without drastic compromises to the other hydraulic functions. Research involving controlled mechanical stimuli and using transgenic trees with modified cell wall structure (e.g., altered lignin or cellulose), may be a promising mean of elucidating mechanisms of xylem construction that may be constrained by the functional trade-offs.

## Conflict of Interest Statement

The authors declare that the research was conducted in the absence of any commercial or financial relationships that could be construed as a potential conflict of interest.

## References

[B1] Alméras T. Costes E. Salles J.-C. (2004). Identification of biomechanical factors involved in stem shape variability between apricot tree varieties. *Ann. Bot.* 93 455–468 10.1093/aob/mch054 14980974 PMC4242333

[B2] Anten N. P. R. Casado-Garcia R. Nagashima H. (2005). Effects of mechanical stress and plant density on mechanical properties, growth, and lifetime reproduction of Tobacco plants. *Am. Nat.* 166 650–660 10.1086/497442 16475082

[B3] Awad H. Herbette H. Brunel N. Tixier A. Pilate G. Cochard H. (2012). No trade-off between hydraulic and mechanical properties in several transgenic poplars modified for lignins metabolism. *Environ. Exp. Bot.* 77 185–195 10.1016/j.envexpbot.2011.11.023

[B4] Baas P. (1986). “Ecological patterns of xylem anatomy,” in *On the Economy of Plant form and Function* ed. Givnish T. J. (Cambridge: Cambridge University Press) 327–351.

[B5] Biddington N. L. (1986). The effects of mechanically-induced stress in plants- a review. *Plant Growth Regul.* 4 103–123 10.1007/BF00025193

[B6] Bouche P. S. Larter M. Domec J. C. Burlett R. Gasson P. Jansen S. (2014). A broad survey of hydraulic and mechanical safety in the xylem of conifers. *J. Exp. Bot.* 65 4419–4431 10.1093/jxb/eru218 24916072 PMC4112641

[B7] Braam J. (2005). In touch: plant responses to mechanical stimuli. *New Phytol.* 165 373–389 10.1111/j.1469-8137.2004.01263.x 15720650

[B8] Butterfield B. G. Li G. (2000). *Wood Properties of Glass House Grown Clonal Radiata Plantlets.* Report to the multiclient seedling group, University of Canterbery 12.

[B9] Carlquist S. (1975). *Ecological Strategies of Xylem Evolution*. Berkeley, CA: University of California Press.

[B10] Cave I. D. Hutt L. (1969). The longitudinal Young’s modulus of *Pinus Radiata*. *Wood Sci. Technol.* 3 40–48 10.1007/BF00349983

[B11] Cave I. D. Walker J. C. F. (1994). Stiffness of wood in fast-grown plantation softwoods: the influence of microfibril angle. *Forest Prod. J.* 44 43–48.

[B12] Chave J. Coomes D. Jansen S. Lewis S. L. Swenson N. G. Zanne A. E. (2009). Towards a worldwide wood economics spectrum. *Ecol. Lett.* 12 351–366 10.1111/j.1461-0248.2009.01285.x 19243406

[B13] Chehab E. W. Eich E. Braam J. (2009). Thigmomorphogenesis: a complex plant response to mechano-stimulation. *J. Exp. Bot.* 60 43–56 10.1093/jxb/ern315 19088336

[B14] Choat B. Ball M. Luly J. Holtum J. (2003). Pit membrane porosity and water stress-induced cavitation in four co-existing dry rainforest tree species. *Plant Physiol.* 131 41–48 10.1104/pp.014100 12529513 PMC166785

[B15] Choat B. Ball M. C. Luly J. G. Holtum J. A. M. (2005). Hydraulic architecture of deciduous and evergreen dry rainforest tree species from north-eastern Australia. *Trees* 19 305–311 10.1007/s00468-004-0392-1

[B16] Christensen-Dalsgaard K. K. Fournier M. Ennos A. R. Barfod A. S. (2007). Changes in vessel anatomy in response to mechanical loading in six species of tropical trees. *New Phytol.* 176 610–622 10.1111/j.1469-8137.2007.02227.x 17953543

[B17] Clair B. Almeras T. Sugiyama J. (2006). Compression stress in opposite wood of angiosperms: observations in chestnut, mani and poplar. *Ann. For. Sci.* 63 507–510 10.1051/forest:2006032

[B18] Cochard H. (1992). Vulnerability of several conifers to air embolism. *Tree Physiol.* 11 73–83 10.1093/treephys/11.1.73 14969968

[B19] Cochard H. Cruiziat P. Tyree M. T. (1992). Use of positive pressures to establish vulnerability curves. Further support for the air-seeding hypothesis and implications for pressure-volume analysis. *Plant Physiol*. 100 205–209 10.1104/pp.100.1.205 16652947 PMC1075538

[B20] Cochard H. Tyree M. T. (1990). Xylem dysfunction in *Quercus*: vessel sizes, tyloses, cavitation and seasonal changes in embolism. *Tree Physiol.* 6 393–408 10.1093/treephys/6.4.393 14972931

[B21] Coleman H. D. Samuels A. L. Guy R. D. Mansfield S. D. (2008). Perturbed lignification impacts tree growth in hybrid poplar-A function of sink strength, vascular integrity, and photosynthetic assimilation. *Plant Physiol.* 148 1229–1237 10.1104/pp.108.125500 18805953 PMC2577275

[B22] Coutand C. (2010). Mechanosensing and thigmomorphogenesis, a physiological and biomechanical point of view. *Plant Sci.* 179 168–182 10.1016/j.plantsci.2010.05.001

[B23] Dalla-Salda G. Fernández M. E. Sergent A. S. Rozenberg P. Badel E. Martinez-Meier A. (2014). Dynamics of cavitation in a Douglas-fir tree-ring: transition-wood, the lord of the ring? *J. Plant Hydraulics* 1:e-0005.

[B24] Davis S. D. Sperry J. S. Hacke U. G. (1999). The relationship between xylem conduit diameter and cavitation caused by freezing. *Am. J. Bot.* 86 1367–1372 10.2307/2656919 10523278

[B25] Dean T. J. (1991). Effects of growth rate and wind sway on the relationship between mechanical and water-flow properties in slash pine seedlings. *Can. J. For. Res.* 21 1501–1506 10.1139/x91-210

[B26] de Langre E. (2008). Effects of wind on plants. *Ann. Rev. Fluid Mech.* 40 141–168 10.1146/annurev.fluid.40.111406.102135

[B27] Dixon H. (1914). *Transpiration and the Ascent of Sap in Plants*. London: Macmillian 216 10.5962/bhl.title.1943

[B28] Domec J. C. Scholz F. G. Bucci S. J. Meinzer F. C. Goldstein G. Villalobos-Vega R. (2006). Diurnal and seasonal variation in root xylem embolism in neotropical savanna woody species: impact on stomatal control of plant water status. *Plant Cell Environ.* 29 26–35 10.1111/j.1365-3040.2005.01397.x 17086750

[B29] Du S. Yamamoto F. (2007). An overview of the biology of reaction wood formation. *J. Integr. Plant Biol.* 49 131–143 10.1111/j.1744-7909.2007.00427.x

[B30] Evans R. (1998). “Rapid scanning of microfibril angles in increments cores by X-ray diffraction,” in *Microfibril Angle in Wood* ed. Butterfield B. G. (Christchurch: University of Canterbury) 116–139.

[B31] Evans R. Elic J. (2001). Rapid prediction of wood stiffness from microfibril angle and density. *Forest Prod. J.* 51 53.

[B32] Fournier M. Stokes A. Coutand C. Fourcaud T. Moulia B. (2006). “Tree biomechanics and growth strategies in the context of forest functional ecology,” in *Ecology and Biomechanics: A Mechanical Approach to the Ecology of Animals and Plants* eds Herrel A. Speck T. Rowe N. (Boca Raton, FL: CRC Press) 1–34 10.2307/2389490

[B33] Gartner B. L. (1991a). Is the climbing habit of poison oak ecotypic? *Funct. Ecol*. 5 696–704 10.2307/2389490

[B34] Gartner B. L. (1991b). Stem hydraulic-properties of vines vs. shrubs of western poison oak *Toxicodendron diversilobum*. *Oecologia* 87 180–189 10.1007/BF00325255 28313834

[B35] Gartner B. L. (1991c). Structural stability and architecture of vines vs. shrubs of poison oak *Toxicodendron diversilobum*. *Oecologia* 72 2005–2015 10.2307/1941555 28313834

[B36] Gartner B. L. Roy J. Huc R. (2003). Effects of tension wood on specific conductivity and vulnerability to embolism of *Quercus ilex* seedlings grown at two atmospheric CO_2_ concentrations. *Tree Physiol.* 23 387–395 10.1093/treephys/23.6.387 12642240

[B37] Grace J. (1977). *Plant Response to Wind*. London: Academic Press.

[B38] Hacke U. G. Sperry J. S. Pockman W. T. Davis S. D. McCulloh K. A. (2001). Trends in wood density and structure are linked to prevention of xylem implosion by negative pressure. *Oecologia* 126 457–461 10.1007/s004420100628 28547229

[B39] Hölttä T. Cochard H. Nikinmaa E. Mencuccini M. (2009). Capacitive effect of cavitation in xylem conduits: results from a dynamic model. *Plant Cell Environ.* 32 10–21 10.1111/j.1365-3040.2008.01894.x 19076529

[B40] Iida Y. Kohyama T. S. Swenson N. G. Su S. H. Chen C. T. Chiang J. M. (2014). Linking functional traits and demographic rates in a subtropical tree community: the importance of size dependency. *J. Ecol.* 102 641–650 10.1111/1365-2745.12221

[B41] Jacobs M. R. (1954). The effect of wind sway on the formand development of *Pinus radiate* D. Don. *Aust. J. Bot.* 2 35–51 10.1071/BT9540035

[B42] Jacobsen A. L. Ewers F. W. Pratt R. B. Paddock W. A. Davis S. D. (2005). Do xylem fibers affect xylem cavitation resistance? *Plant Physiol*. 139 546–556 10.1104/pp.104.058404 16100359 PMC1203402

[B43] Jaffe M. J. (1973). Thigmomorphogenesis: the response of plant growth and development to mechanical stimulation. *Planta* 114 143–157 10.1007/BF00387472 24458719

[B44] Jaffe M. J. (1985). “Wind and other mechanical effects in the development and behaviour of plants, with special emphasis on the role of hormones,” in *Hormonal Regulation of Development Vol. 11, III Role of Environmental Factors, Encyclopedia of Plant Physiology, NS* eds Pharis R. P. Reid D. M. (Berlin: Springer) 444–483.

[B45] Jaffe M. J. Leopold A. C. Staples R. A. (2002). Thigmo responses in plants and fungi. *Am. J. Bot.* 89 375–382 10.3732/ajb.89.3.375 21665632

[B46] Jagels R. Visscher G. E. Lucas J. Goodell B. (2003). Paleo-adaptive properties of the xylem of *Metasequoia*: mechanical/hydraulic compromises. *Ann. Bot.* 92 79–88 10.1093/aob/mcg117 12763758 PMC4243641

[B47] Jones L. Ennos A. R. Turner S. R. (2001). Cloning and characterization of irregular xylem4 (irx4): a severely lignin-deficient mutant of *Arabidopsis*. *Plant J.* 2 205–216 10.1046/j.1365-313x.2001.01021.x 11389761

[B48] Kern K. A. Ewers F. W. Telewski F. W. Koehler L. (2005). Mechanical perturbation affects conductivity, mechanical properties and aboveground biomass of hybrid poplars. *Tree Physiol.* 25 1243–1251 10.1093/treephys/25.10.1243 16076773

[B49] Knight T. A. (1803). Account of some experiments on the decent of sap in trees. *Philos. Trans. R. Soc.* 96 277–289 10.1098/rstl.1803.0011

[B50] Koehler L. Telewski F. W. (2006). Biomechanics and transgenic wood. *Am. J. Bot.* 93 1433–1438 10.3732/ajb.93.10.1433 21642090

[B51] Lachenbruch B. McCulloh K. A. (2014). Traits, properties, and performance: how woody plants combine hydraulic and mechanical functions in a cell, tissue, or whole plant. *New Phytologist.* 204 747–764 10.1111/nph.13035 25250668

[B52] Larjavaara M. (2010). Maintenance cost, toppling risk and size of trees in a self-thinning stand. *J. Theor. Biol.* 265 63–67 10.1016/j.jtbi.2010.04.021 20417645

[B53] Liu X. Silins U. Lieffers V. J. Man R. (2002). Stem hydraulic properties and growth in lodgepole pine stands following thinning and sway treatment. *Can. J. For. Res.* 33 1295–1303 10.1139/x03-061

[B54] Liu X. Swenson N. G. Wright S. J. Zhang L. Song K. Du Y. (2012). Covariation in plant functional traits and soil fertility within two species-rich forests. *PLoS ONE* 7:e34767 10.1371/journal.pone.0034767 PMC3318000 22509355

[B55] Liu X. Swenson N. G. Zhang J. Ma K. (2013). The environment and space, not phylogeny, determine trait dispersion in a subtropical forest. *Funct. Ecol.* 27 264–272 10.1111/1365-2435.12018

[B56] Martin L. Leblanc-Fournier N. Julien J. L. Moulia B. Coutand C. (2010). Acclimation kinetics of physiological and molecular responses of plants to multiple mechanical loadings. *J. Exp. Bot.* 61 2403–2412 10.1093/jxb/erq069 20363866

[B57] Martinez-Vilalta J. Prat E. Oliveras I. Pinol J. (2002). Xylem hydraulic properties of roots and stems of nine Mediterranean woody species. *Oecologia* 133 19–29 10.1007/s00442-002-1009-2 24599365

[B58] Mayr S. Cochard H. (2003). A new method for vulnerability analysis of small xylem areas reveals that compression wood of Norway spruce has lower hydraulic safety than opposite wood. *Plant Cell Environ.* 26 1365–1371 10.1046/j.0016-8025.2003.01060.x

[B59] Mayr S. Hacke U. G. Schmid P. Schwienbacher F. Gruber A. (2006). Frost drought in conifers at the alpine timberline: xylem dysfunction and adaptations. *Ecology* 87 3175–3185 10.1890/0012-9658(2006)87[3175:FDICAT]2.0.CO;2 17249241

[B60] McMahon T. A. (1973). Size and shape in biology. *Science* 179 1201–1204 10.1126/science.179.4079.1201 4689015

[B61] Meinzer F. C. Johnson D. M. Lachenbruch B. McCulloh K. A. Woodruff D. R. (2009). Xylem hydraulic safety margins in woody plants: coordination of stomatal control of xylem tension with hydraulic capacitance. *Funct. Ecol.* 23 922–930 10.1111/j.1365-2435.2009.01577.x

[B62] Mitchell C. A. (1996). Recent advances in plant responses to mechanical stress: theory and application. *Hort Science* 31 31–35. 11539195

[B63] Mitchell S. J. (2013). Wind as a natural disturbance agent in forests: a synthesis. *Forestry* 86 147–157 10.1093/forestry/cps058

[B64] Moulia B. Coutand C. Julien J. L. (2015). Mechanosensitive control of plant growth: bearing the load, sensing, transducing, and responding. *Front. Plant Sci.* 6:52 10.3389/fpls.2015.00052 PMC4337334 25755656

[B65] Moulia B. Coutand C. Lenne C. (2006). Posture control and skeletal mechanical acclimation in terrestrial plants: implications for mechanical modeling of plant architecture. *Am. J. Bot.* 93 1477–1489 10.3732/ajb.93.10.1477 21642095

[B66] Neel P. L. Harris R. W. (1971). Motion-induced inhibition of elongation and induction of dormancy in *Liquidambar*. *Science* 173 58–59 10.1126/science.173.3991.58 17747314

[B67] Niklas K. J. (1992). *Plant Biomechanics: An Engineering Approach to Plant form and Function*. Chicago: University of Chicago Press.

[B68] Niklas K. J. (1994). Interspecific allometries of critical buckling height and actual plant height. *Am. J. Bot.* 81 1275–1279 10.2307/2445403

[B69] Pittermann J. Sperry J. (2003). Tracheid diameter is the key trait determining the extent of freezing-induced embolism in conifers. *Tree Physiol.* 23 907–914 10.1093/treephys/23.13.907 14532014

[B70] Pittermann J. Sperry J. S. Wheeler J. K. Hacke U. G. Sikkema E. H. (2006). Mechanical reinforcement of tracheids compromises the hydraulic efficiency of conifer xylem. *Plant Cell Environ.* 29 1618–1628 10.1111/j.1365-3040.2006.01539.x 16898022

[B71] Poorter L. (2008). The relationships of wood-, gas- and water fractions of tree stems to performance and life history variation in tropical trees. *Ann. Bot*. 102 367–375 10.1093/aob/mcn103 18573862 PMC2701806

[B72] Poorter L. McDonald I. Alarcón A. Fichtler E. Licona J. C. Peña-Claros M. (2010). The importance of wood traits and hydraulic conductance for the performance and life history strategies of 42 rainforest tree species. *New Phytol.* 185 481–492 10.1111/j.1469-8137.2009.03092.x 19925555

[B73] Pratt R. B. Jacobsen A. L. Ewers F. W. Davis S. D. (2007). Relationships among xylem transport, biomechanical and storage in stems and roots of nine Rhamnaceae species of the California chaparral. *New Phytol.* 174 787–798 10.1111/j.1469-8137.2007.02061.x 17504462

[B74] Pruyn M. Ewers B. J. Telewski F. W. (2000). Thigmomorphogenesis: change in the morphology and mechanical properties of two *Populus* hybrids in response to mechanical perturbation. *Tree Physiol.* 20 535–540 10.1093/treephys/20.8.535 12651434

[B75] Rosner S. Klein A. Muller U. Karlsson B. (2007). Hydraulic and mechanical properties of young Norway spruce clones related to growth and wood structure. *Tree Physiol.* 27 1165–1178 10.1093/treephys/27.8.1165 17472942 PMC3197722

[B76] Rosner S. Klein A. Muller U. Karlsson B. (2008). Tradeoffs between hydraulic and mechanical stress responses of mature Norway spruce trunk wood. *Tree Physiol.* 28 1179–1188 10.1093/treephys/28.8.1179 18519249 PMC3196968

[B77] Rudnicki M. Mitchell S. J. Novak M. D. (2004). Wind tunnel measurements of crown streamlining and drag relationships for three conifer species. *Can. J. For. Res.* 34 666–676 10.1139/x03-233

[B78] Smith V. C. Ennos A. R. (2003). The effects of air flow and stem flexure on the mechanical and hydraulic properties of the stems of sunflowers *Helianthus annuus* L. *J. Exp. Bot.* 54 845–849 10.1093/jxb/erg068 12554727

[B79] Sperry J. S. (2003). Evolution of water transport and xylem structure. *Int. J. Plant Sci.* 164 115–127 10.1086/368398

[B80] Sperry J. S. Hacke U. G. (2004). Analysis of circular bordered pit function. I. Angiosperm vessels with homogenous pit membranes. *Am. J. Bot.* 91 369–385 10.3732/ajb.91.3.369 21653393

[B81] Sperry J. Hacke U. G. Pittermann J. (2006). Size and function in conifer tracheids and and angiosperm vessels. *Am. J. Bot.* 93 1490–1500 10.3732/ajb.93.10.1490 21642096

[B82] Sperry J. S. Nichols K. L. Sullivan J. E. M. Eastlack S. E. (1994). Xylem embolism in ring-porous, diffuse-porous, and coniferous trees of Northern Utah and Interior Alaska. *Ecology* 75 1736–1752 10.2307/1939633

[B83] Sperry J. S. Tyree M. T. (1988). Mechanism of water stress-induced xylem embolism. *Plant Physiol.* 88 581–587 10.1104/pp.88.3.581 16666352 PMC1055628

[B84] Spicer R. Gartner B. L. (1998). Hydraulic properties of douglas-fir (*Pseudotsuga menziesii*) branches and branch halves with reference to compression wood. *Tree Physiol*. 18 777–784 10.1093/treephys/18.11.777 12651412

[B85] Stegen J. C. Swenson N. G. Valencia R. Enquist B. J. Thompson J. (2009). Above-ground forest biomass is not consistently related to wood density in tropical forests. *Global Ecol. Biogeogr.* 18 617–625 10.1111/j.1466-8238.2009.00471.x

[B86] Swenson N. G. Anglada-Cordero P. Barone J. A. (2011). Deterministic tropical tree community turnover: evidence from patterns of functional beta diversity along an elevational gradient. *Proc. R. Soc. B Biol. Sci.* 278 877–884 10.1098/rspb.2010.1369 PMC3049044 20861048

[B87] Swenson N. G. Weiser M. D. (2010). Plant geography upon the basis of functional traits: an example from eastern North American trees. *Ecology* 91 2234–2241 10.1890/09-1743.1 20836445

[B88] Telewski F. W. (1989). Structure and function of flexure wood in *Abies fraseri*. *Tree Physiol.* 5 113–121 10.1093/treephys/5.1.113 14973003

[B89] Telewski F. W. (1995). “Wind induced physiological and developmental responses in trees,” in *Wind and Trees* eds Coutts M. P. Grace J. (Cambridge: Cambridge University Press) 237–263.

[B90] Telewski F. W. (2006). A unified hypothesis of mechanoperception in plants. *Am. J. Bot.* 93 1306–1316 10.3732/ajb.93.10.1466 21642094

[B91] Telewski F. W. (2012). Is windswept tree growth negative Thigmotropism? *Plant Sci.* 184 20–28 10.1016/j.plantsci.2011.12.001 22284706

[B92] Telewski F. W. Aloni R. Sauter J. (1996). “Physiology of Secondary Tissues of Populus,” in *Biology of Populus and its Implications for Management and Conservation, Part II. Physiology of Growth, Productivity and Stress Responses* eds Stettler R. F. Bradshaw H. D. Jr. Heilman P. E. Hinckley T. M. (Ottawa: NCR Research Press) 301–329 10.1111/j.1399-3054.1986.tb02411.x

[B93] Telewski F. W. Jaffe M. J. (1986a). Thigmomorphogenesis: field and laboratory studies of *Abies fraseri* in response to wind or mechanical perturbation. *Physiol. Plant.* 66 211–218 10.1111/j.1399-3054.1986.tb02411.x 11538654

[B94] Telewski F. W. Jaffe M. J. (1986b). Thigmomorphogenesis: anatomical, morphological and mechanical analysis of genetically different sibs of *Pinus taeda* L. in response to mechanical perturbation. *Physiol. Plant.* 66 219–226 10.1111/j.1399-3054.1986.tb02412.x 11538655

[B95] Telewski F. W. Pruyn M. (1998). Thigmomorphogenesis: a dose response to flexing in *Ulmus americana* seedlings. *Tree Physiol.* 18 65–68 10.1093/treephys/18.1.65 12651301

[B96] Timell T. E. (1986a). *Compression Wood in Gymnosperms* Vol. 1 Berlin: Springer-Verlag.

[B97] Timell T. E. (1986b). *Compression Wood in Gymnosperms* Vol. 2 Berlin: Springer-Verlag.

[B98] Timell T. E. (1986c). *Compression Wood in Gymnosperms*, Vol. 3 Berlin: Springer-Verlag.

[B99] Tixier A. Herbette S. Jansen S. Capron M. Tordjeman P. Cochard H. (2014). Modelling the mechanical behaviour of pit membranes in bordered pits with respect to cavitation resistance in angiosperms. *Ann. Bot.* 114 325–334 10.1093/aob/mcu109 24918205 PMC4111388

[B100] Turner S. R. Somerville C. R. (1997). Collapsed xylem phenotype of *Arabidopsis* identifies mutants deficient in cellulose deposition in the secondary cell wall. *Plant Cell* 9 689–701 10.1105/tpc.9.5.689 9165747 PMC156949

[B101] Tyree M. T. Davis S. D. Cochard H. (1994). Biophysical perspectives of xylem evolution: is there a trade-off of hydraulic efficiency for vulnerability to dysfunction? *IAWA J.* 15 335–360 10.1163/22941932-90001369

[B102] Tyree M. T. Velez V. Dalling J. W. (1998). Growth dynamics of root and shoot hydraulic conductance in seedlings of five neotropical tree species. Scaling to show possible adaptation to differing light regimes. *Oecologia* 114 293–298 10.1007/s004420050450 28307771

[B103] Tyree M. T. Zimmermann M. H. (2002). *Xylem Structure and the Ascent of Sap* 2nd Edn Berlin: Springer 10.1007/978-3-662-04931-0

[B104] Utsumi Y. Bobich E. G. Ewers F. W. (2010). Photosynthetic, hydraulic and biome-chanical responses of *Juglans californica* shoots to wildfire. *Oecologia* 164 331–338 10.1007/s00442-010-1653-x 20496153

[B105] Vaast P. Angrand J. Ffranck N. Dauzat J. Génard M. (2005). Fruit load and branch ring-barking affect carbon allocation and photosynthesis of leaf and fruit of *Coffea arabica* in the field. *Tree Physiol.* 25 753–760 10.1093/treephys/25.6.753 15805095

[B106] Valinger E. Lundquist L. Sundberg B. (1995). Mechanical bending stress applied during dormancy and (or) growth stimulates stem diameter growth of scots pine-seedlings. *Can. J. For. Res.* 25 886–890 10.1139/x95-097

[B107] Vogel S. (1994). *Life in Moving Fluids: The Physical Biology of Flow*. 2nd Edn Princeton, NJ: Princeton University Press.

[B108] Vollsinger S. Mitchell S. J. Byrne K. E. Novak M. D. Rudnicki M. (2005). Wind tunnel measurements of crown streamlining and drag relationships for several hardwood species. *Can. J. For. Res.* 35 1238–1249 10.1139/x05-051

[B109] Wagner K. R. Ewers F. W. Davis S. D. (1998). Tradeoffs between hydraulic efficiency and mechanical strength in the stems of four co-occurring species of chaparral shrubs. *Oecologia* 117 53–62 10.1007/s004420050631 28308506

[B110] Wainwright S. A. Briggs W. D. Currey J. D. Gosline J. M. (1976). *Mechanical Design in Organisms*. New York, NY: John Wiley and Sons.

[B111] Wheeler J. K. Sperry J. S. Hacke U. G. Hoang N. (2005). Inter-vessel pitting and cavitation in woody Rosaceae and other vesselled plants: a basis for a safety versus efficiency trade-off in xylem transport. *Plant Cell Environ.* 28 800–813 10.1111/j.1365-3040.2005.01330.x

[B112] Wilson B. F. Archer R. R. (1977). Reaction wood: inducvtion and mechanical action. *Ann. Rev. Plant Physiol.* 28 23–43 10.1146/annurev.pp.28.060177.000323

[B113] Woodrum C. L. Ewers F. W. Telewski F. W. (2003). Hydraulic, biomechanical, and anatomical interactions of xylem from five species of Acer (Aceraceae). *Am. J. Bot.* 90 693–699 10.3732/ajb.90.5.693 21659164

[B114] Yamamoto H. Ruelle J. Arakawa Y. Yoshida M. Clair B. Gril J. (2010). Origin of the characteristic hygro-mechanical properties of the gelatinous layer in tension wood from Kunugi oak (*Quercus acutissima*) *Wood Sci. Technol*. 44 149–163 10.1007/s00226-009-0262-5

[B115] Zanne A. E. Westoby M. Falster D. S. Ackerly D. D. Loarie S. R. Arnold S. E. (2010). Angiosperm wood structure: global patterns in vessel anatomy and their relation to wood density and potential conductivity. *Am. J. Bot.* 97 207–215 10.3732/ajb.0900178 21622380

[B116] Zimmermann M. H. (1983). *Xylem Structure, and the Ascent of Sap*. Berlin: Springer-Verlag 10.1007/978-3-662-22627-8

